# Multiple steps of dynein activation by Lis1 visualized by cryo-EM

**DOI:** 10.1038/s41594-025-01558-w

**Published:** 2025-05-23

**Authors:** Agnieszka A. Kendrick, Kendrick H. V. Nguyen, Wen Ma, Eva P. Karasmanis, Rommie E. Amaro, Samara L. Reck-Peterson, Andres E. Leschziner

**Affiliations:** 1 Salk Institute for Biological Studies, La Jolla, CA USA; 2 Department of Cellular and Molecular Medicine, University of California San Diego, La Jolla, CA USA; 3 Department of Physics, University of Vermont, Burlington, VT USA; 4 Department of Chemistry and Biochemistry, University of California San Diego, La Jolla, CA USA; 5 Department of Cell and Developmental Biology, University of California San Diego, La Jolla, CA USA; 6 Howard Hughes Medical Institute, Chevy Chase, MD USA; 7 Department of Molecular Biology, University of California San Diego, La Jolla, CA USA

**Keywords:** Cryoelectron microscopy, Dynein, Motor protein structure

## Abstract

Cytoplasmic dynein-1 (dynein) is an essential molecular motor controlled in part by autoinhibition. Lis1, a key dynein regulator mutated in the neurodevelopmental disease lissencephaly, plays a role in dynein activation. We recently identified a structure of partially autoinhibited dynein bound to Lis1, which suggests an intermediate state in dynein’s activation pathway. However, other structural information is needed to fully understand how Lis1 activates dynein. Here, we used cryo-EM and yeast dynein and Lis1 incubated with ATP at different time points to reveal conformations that we propose represent additional intermediate states in dynein’s activation pathway. We solved 16 high-resolution structures, including 7 distinct dynein and dynein–Lis1 structures from the same sample. Our data support a model in which Lis1 relieves dynein autoinhibition by increasing its basal ATP hydrolysis rate and promoting conformations compatible with complex assembly and motility. Together, this analysis advances our understanding of dynein activation and the contribution of Lis1 to this process.

## Abstract (web summary)

Using cryo-EM, Kendrick et al. reveal multiple dynein conformations during dynein’s mechanochemical cycle and Lis1 binding that represent intermediate states of dynein’s activation.

## Main

Cytoplasmic dynein-1 (dynein) is a highly conserved molecular motor that moves toward the microtubule minus end, transporting various cargoes including RNAs, membrane vesicles, and viruses, and plays a crucial role in cell division^1^. The dynein complex (1.4 MDa) comprises a dimer of two motor domains and two copies each of five accessory chains (an intermediate chain, a light intermediate chain, and three light chains)^1,2^. Cellular and structural work suggests that dynein exists predominantly in the autoinhibited Phi conformation in cells^3–5^. Once released from autoinhibition, dynein undergoes conformational changes to assemble into an active complex containing one or two dynein dimers and its cofactors: a multisubunit dynactin complex (1.1 MDa) and a coiled-coil activating adapter that also links the active complex to cargo^1,6,7^. Mutations in dynein and its binding partners are linked to neurodevelopmental and neurodegenerative diseases^8^.

**Fig. 1:**
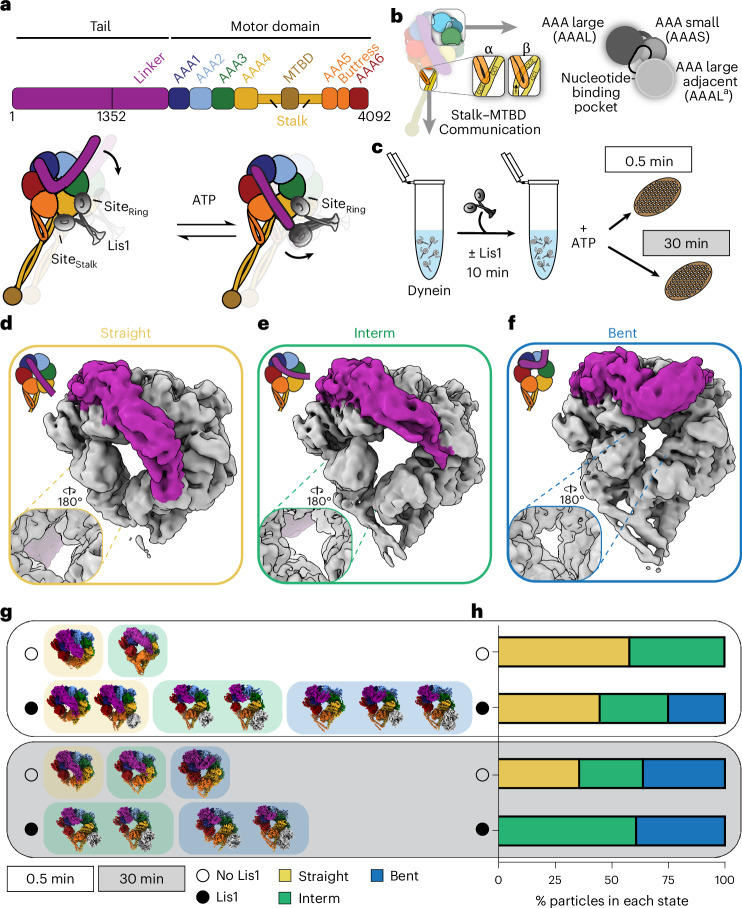
Time-resolved cryo-EM captures Lis1’s effect on dynein’s conformational landscape during ATP hydrolysis. **a**, Schematic of dynein domain organization. Lis1 binding at different sites on dynein (site_ring_ and site_stalk_) is shown in different dynein conformations^43^. MTBD, microtubule-binding domain. **b**, The architecture of a nucleotide-binding pocket and Stalk–MTBD communication. **c**, Schematic of the experimental setup. **d**–**f**, The three major groups of conformations identified in the datasets are shown on locally refined cryo-EM volumes, filtered to 6 Å: straight linker (**d**), intermediate linker (**e**), and bent linker (**f**). The linker region is highlighted in magenta. The extent of ring opening is shown to the left of each panel. **g**, Different conformations were identified in each dataset in the absence (open circle) or presence of Lis1 (black circle) at two different time points (0.5 min, white background; 30 min, gray background). **h**, Relative abundance of particles belonging to different states obtained from particle distributions in cryo-EM datasets in the absence (open circle) or presence of Lis1 (black circle) at two different time points (0.5 min, white background; 30 min, gray background). Source data

Dynein belongs to the ATPase associated with various cellular activities (AAA+) family of proteins. Its heavy chain features a motor domain composed of six AAA+ modules, a long stalk, a microtubule-binding domain (MTBD) located at the end of the stalk, a buttress region that makes additional connections between the AAA5 module and the stalk, and a long tail that interacts with dynactin and an activating adapter (Fig. 1a). The tail also generates a power stroke through its mechanical element, called the linker^9^. Among the six AAA+ modules, four bind ATP (AAA1, AAA2, AAA3, and AAA4), but only three hydrolyze it (AAA1, AAA3, and AAA4)^10–14^. AAA5 and AAA6 do not contain the necessary residues for nucleotide binding^12,14^. Each AAA module is composed of a large and small domain, with nucleotide binding taking place in a groove between them and the large domain of the adjacent AAA module (Fig. 1b).

Dynein harnesses the chemical energy of ATP to generate movement and force through its mechanochemical cycle^15^. This process involves ATP binding and hydrolysis in the primary catalytic module, AAA1, which triggers large conformational changes that lead to the opening and closing of the AAA ring, rearrangement of the linker to generate the power stroke, and reorganization of helices in the buttress and stalk (CC1 and CC2) to regulate dynein’s interaction with microtubules through the MTBD (Fig. 1a,b)^11,16,17^. This reorganization is due to the sliding of the CC1 with respect to CC2. The helices can adopt two main ‘registers,’ referred to as α and β, which correspond to high and low microtubule-binding affinities, respectively (Fig. 1b)^18,19^. Mutations in AAA3 that prevent it from hydrolyzing ATP also slow down ATP hydrolysis in AAA1, providing evidence for an allosteric role for AAA3 in regulating dynein’s mechanochemistry^11,17,20^.

Dynein function in vivo is tightly linked to Lis1, the only dynein regulator that binds directly to dynein’s motor domain^21–23^. Lis1 is a dimer (~90 kDa) with an amino-terminal dimerization domain and carboxy-terminal β-propellers that bind to the dynein motor domain^24,25^. Human LIS1 was initially identified as a gene (*PAFAH1B1*) that is mutated in the neurodevelopmental disease lissencephaly, which is also linked to mutations in dynein’s heavy chain (*DYNC1H1*)^8,26,27^. Lis1 is conserved from fungi, including *Saccharomyces* *cerevisiae*, to humans. Dynein is important for mitotic spindle positioning in yeast, although it is non-essential in that organism, providing an excellent model system to study dynein function^28–32^. Like human dynein, yeast dynein requires dynactin and the presumed activating adapter Num1 for function^29–31,33^. Work in yeast and other organisms has shown that Lis1 is important for forming active dynein complexes^34–38^. Recently, we showed that Lis1 does so by relieving dynein’s autoinhibited Phi conformation. We found that two Lis1 dimers could disrupt the Phi conformation by wedging themselves between two dynein protomers (an intermediate state called Chi), potentially priming dynein for subsequent complex assembly and activation^39^. Lis1 has a crucial role in the dynein activation pathway by directly interacting with dynactin to support conformations involved in complex assembly^40^. In addition, the dynein–dynactin–activating adapter complexes assembled in the presence of Lis1 recruit an additional dynein dimer, and these complexes move faster on microtubules^34,36^. Taken together, these findings suggest that Lis1 has multiple roles in regulating dynein function that could go beyond Phi particle opening. Indeed, mutations in dynein that block the formation of Phi do not fully rescue phenotypes resulting from Lis1 disruption^38,39,41,42^. Thus, more structural and mechanistic studies are needed to understand how Lis1 regulates dynein.

Lis1 binds to dynein at three sites, one between AAA3 and AAA4 (site_ring_), one in the stalk region (site_stalk_) (Fig. 1a), and a third one that is observed in the presence of a second dynein protomer in the Chi conformation, in *trans* at AAA5 and AAA6 (refs. ^22,35,39,43,44^). Previous analysis showed that the nucleotide state at AAA3 regulates Lis1’s association with dynein and dynein’s microtubule-binding state^43^. However, recent findings suggest that Lis1 has crucial roles in dynein activation and complex assembly, pointing to a mechanism by which Lis1 regulates dynein before dynein interacts with microtubules. Indeed, a dynein mutant locked in a microtubule-unbound state binds to Lis1 with a stronger affinity than does its microtubule-bound counterpart, consistent with data showing that Lis1 can dissociate from active dynein complexes bound to microtubules^34,36,45^. Additionally, a cryogenic electron microscopy (cryo-EM) structure of two dynein dimers in complex with dynactin and an activating adapter (JIP3) revealed that Lis1 binds to the dynein dimer detached from microtubules, whereas the microtubule-bound dynein dimer was not associated with Lis1 (ref. ^40^). Although the authors also suggested that Lis1 changes the nucleotide state of the AAA3 domain, it is unclear what role Lis1 has in the regulation of dynein’s motor activity. Numerous lines of evidence suggest that Lis1 does not affect dynein’s microtubule-stimulated ATPase activity, but how Lis1 effects dynein’s basal ATPase activity, that is when dynein is not bound to microtubules, and during dynein activation remains unclear^21,22,44,46,47^.

To address the gaps in our understanding of dynein’s mechanochemistry and its regulation by Lis1, we used cryo-EM combined with a time-resolved sample-preparation pipeline in the presence of ATP to capture as many detectable states of dynein as we could throughout its mechanochemical cycle (Fig. 1c). Using wild-type dynein monomer and wild-type Lis1 from *S. cerevisiae*, we solved 16 dynein and dynein–Lis1 structures, including previously unknown states. Our data show that Lis1 effects dynein’s conformational landscape by increasing its basal ATPase rate, a finding that has previously been reported but not fully explored^22,44^. Our data support a hypothesis in which Lis1 promotes dynein conformations conducive to transitioning from an autoinhibited state to an open conformation by facilitating a nucleotide exchange in AAA3, potentially priming dynein for complex assembly. As this model predicts, opening the Phi particle through Lis1 binding or by mutating a dynein residue important for Phi formation (p.D2868K) increases dynein’s basal ATPase rate^41^. Taken together, our analysis suggests that dynein’s ATPase activity is important for its activation, and that, by binding to dynein, Lis1 assists dynein’s transition into a state primed for subsequent complex assembly with dynactin and an activating adapter.

## Results

### Cryo-EM reveals different dynein conformations in the presence of Lis1 and ATP

**Extended Data Fig. 1:**
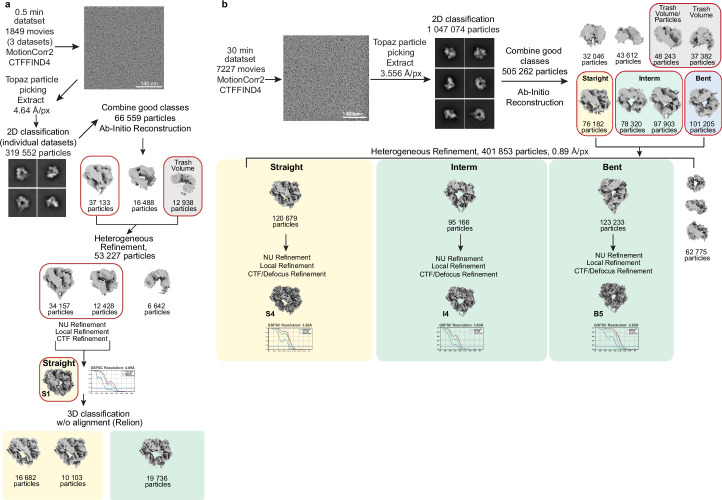
Cryo-EM data processing workflow for the dynein datasets. **a**. Dose-weighted movies from 3 datasets of dynein without Lis1 (0.5 min condition) were aligned with MotionCor2 and CTF was estimated using CCTFFIND4. Particle extraction (binned by 4) was performed in cryoSPARC with a Topaz-trained model. Good particles from 2D classification jobs were used for ab-inito model generation in cryoSPARC. The models in red boxes were carried into the next steps. Fourier shell correlation (FSC) plots are shown next to the final maps. **b**. Dose-weighted movies from a dataset of dynein without. Lis1 (30 min condition) were aligned with MotionCor2 and CTF was estimated using CCTFFIND4. Particle extraction (binned by 4) was performed in cryoSPARC with a Topaz- trained model. Good particles from 2D classification jobs were used for ab-inito model generation in cryoSPARC. The models in red boxes were carried into the next steps. Fourier shell correlation (FSC) plots are shown next to the final maps.

**Extended Data Fig. 2:**
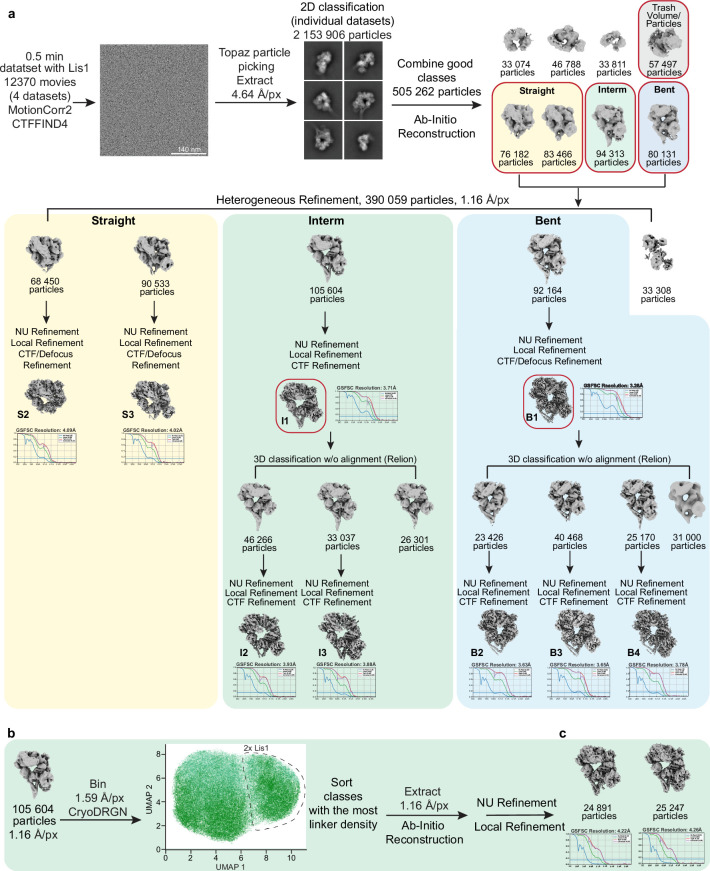
Cryo-EM data processing workflow for the dynein + Lis1 + ATP 0.5 min dataset. **a**. Dose-weighted movies from 4 datasets of dynein with Lis1 (0.5 min condition) were aligned with MotionCor2 and CTF was estimated using CCTFFIND4. Particle extraction (binned by 4) was performed in cryoSPARC with a Topaz-trained model. Good particles from 2D classification jobs were used for ab-inito model generation in cryoSPARC. The models in red boxes were carried into the next steps. Fourier shell correlation (FSC) plots are shown next to the final maps. **b**. The heterogeneity analysis (cryoDRGN) workflow with particles belonging to the intermediate linker dynein (I1) conformation. A UMAP visualization after analysis is shown and particles that show binding of two Lis1 β-propellers are grouped into the highlighted population (2x Lis1). **c**. The two maps used to determine the linker position for the intermediate linker dynein with 1x Lis1 bound (left) and 2x Lis1 bound (right).

**Extended Data Fig. 3:**
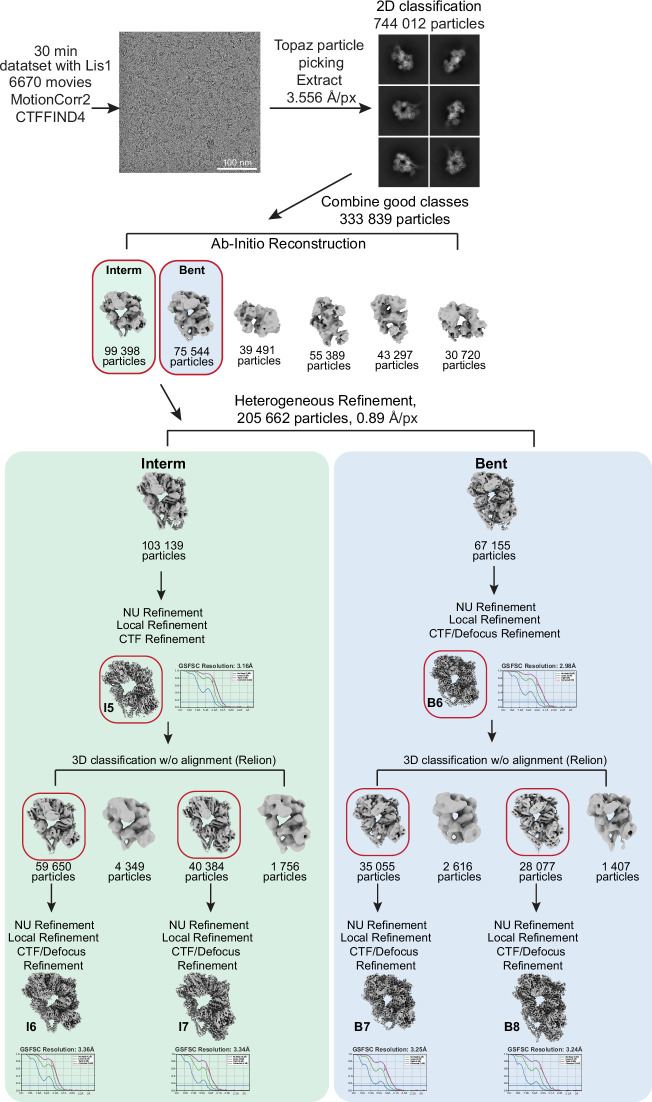
Cryo-EM data processing workflow for the dynein + Lis1 + ATP 30 min dataset. Dose-weighted movies from a dataset of dynein with Lis1 (30 min condition) were aligned with MotionCor2 and CTF was estimated using CCTFFIND4. Particle extraction (binned by 4) was performed in cryoSPARC with a Topaz-trained model. Good particles from 2D classification jobs were used for ab-inito model generation in cryoSPARC. The models in red boxes were carried into the next steps. Fourier shell correlation (FSC) plots are shown next to the final maps.

**Extended Data Fig. 4:**
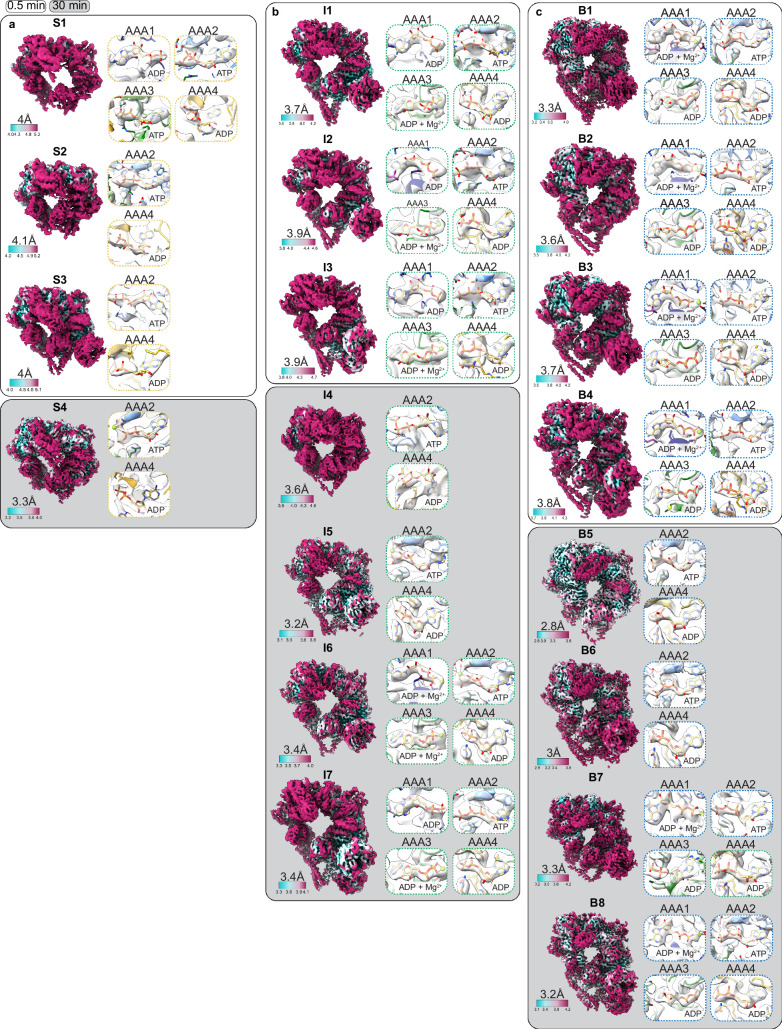
Local resolution and nucleotide occupancies at AAA+ subunits. Local resolution and views of the nucleotide-binding pockets for the indicated AAA+ modules for **a**. straight (S), **b**. intermediate (I), and **c**. bent (B) linker dynein. Conformations belonging to each category are named with the first letter of that category and are shown in order starting with states identified in the shorter time point (0.5 min, white background) followed by the longer time point (30 min, gray background).

Previous high-resolution structures of dynein in complex with Lis1 revealed the interactions between these proteins. However, these structures were solved using mutations or non-hydrolyzable ATP analogs, limiting the ability to capture dynein’s structural dynamics^35,39,43,48^. To characterize the conformational landscape of dynein during Lis1 binding and ATP hydrolysis, we used a time-resolved freezing approach. We purified wild-type yeast dynein monomer (amino acids 1352–4092) in the absence of any nucleotide, plunge-froze grids after the addition of excess ATP (500-fold) at two time points (0.5 min and 30 min) incubating the samples at 4 °C to slow nucleotide hydrolysis. We also prepared grids with samples that were pre-incubated with Lis1 before ATP addition (Fig. 1c). After data collection and analysis (Extended Data Figs. 1–4 and Tables 1 and 2), we observed the expected high level of heterogeneity in our datasets. We grouped the reconstructions into three distinct conformations on the basis of the position of dynein’s linker, a mechanical element that adopts different conformations in response to the ATP-hydrolysis-driven opening and closing of the ring: ‘straight’ (S, yellow outline, Fig. 1d), ‘interm’ (intermediate, I, green outline, Fig. 1e), and ‘bent’ (B, blue outline, Fig. 1f). We named all our maps with letters corresponding to the different conformational states and numbers starting with the shorter time point (0.5 min) (Extended Data Figs. 1–4 and Tables 1 and 2).

**Table 1:** Cryo-EM data collection, refinement and validation statistics for the 0.5 min datasets

Name	S1	S2	S3	I1	I2	I3	B1	B2	B3	B4
State	Straight	Straight	Straight/Lis1	Interm./Lis1	Interm./1×Lis1	Interm./2×Lis1	Bent/Lis1	Bent	Bent/1×Lis1	Bent/2×Lis1
Condition (Lis1)	n/a	Lis1	Lis1	Lis1	Lis1	Lis1	Lis1	Lis1	Lis1	Lis1
Time point (min)	0.5	0.5	0.5	0.5	0.5	0.5	0.5	0.5	0.5	0.5
EMD	EMD-46919	EMD-46897	EMD-46938	EMD-47019	EMD-46958	EMD-46941	EMD-46962	EMD-46940	EMD-46942	EMD-46935
PDB	9DIU	9DI3	9DJU	9DMW	9DKH	9DJZ	9DKM	9DJY	9DK0	9DJ7
**Data collection and processing**										
Magnification	×36,000									
Voltage (kV)	200									
Electron exposure (e^–^/Å^2^)	Dataset 1, 54 Dataset 2, 54 Dataset 3, 55	Dataset 1, 53 Dataset 2, 54 Dataset 3, 54 Dataset 4, 51								
Defocus range (μm)	0.8–2.4									
Pixel size (Å)	1.16									
Symmetry imposed	*C*_1_									
Images (no.)	1,849	12,370								
Initial particles (no.)	319,552	1,047,074								
Final particles (no.)	46,585	68,450	68,486	105,604	46,266	33,037	92,164	23,426	40,468	25,170
Map resolution (Å) (FSC 0.143)	4	4.1	4	3.7	3.9	3.9	3.3	3.6	3.7	3.8
**Refinement**										
Initial model used (PDB code)	7MGM	7MGM	7MGM	7MGM	7MGM	7MGM	7MGM	7MGM	7MGM	7MGM
Model resolution (Å) (FSC 0.5)	4.5	4.3	4.4	4	4.2	4.2	3.4	3.9	3.9	4
Map sharpening *B* factor (Å^2^)	86.5	106	109.8	97.4	85.1	67.4	83.9	63	72.6	60.8
Model composition										
Non-hydrogen atoms	17,847	17,694	20,676	21,212	21,004	23,913	21,339	18,424	21,050	23,851
Protein residues	2,302	2,282	2,643	2,729	2,691	3,047	2,748	2,341	2,690	3,026
Ligands	ADP: 3, ATP: 1	ADP: 3, ATP: 1	ADP: 3, ATP: 1	MG: 1, ADP: 3, ATP: 1	MG: 1, ADP: 3, ATP: 1	MG: 1, ADP: 3, ATP: 1	MG: 1, ADP: 3, ATP: 1	MG:1, ADP:3, ATP:1	MG: 1, ADP: 3, ATP: 1	MG: 1, ADP: 3, ATP: 1
*B* factors (Å^2^)										
Protein	166.53	124.09	140.15	69.90	107.18	141.67	54.14	98.06	71.21	94.99
Ligand	162.06	92.88	70.03	43.58	69.38	118.25	25.38	80.48	46.11	76.40
R.m.s. deviations										
Bond lengths (Å)	0.003	0.004	0.003	0.003	0.003	0.003	0.003	0.003	0.003	0.003
Bond angles (°)	0.715	0.680	0.740	0.701	0.647	0.616	0.562	0.585	0.602	0.613
**Validation**										
MolProbity score	1.51	1.75	1.68	1.40	1.52	1.55	1.37	1.30	1.44	1.51
Clashscore	7.75	9.34	8.46	5.16	6.31	7.23	4.93	4.81	5.96	6.48
Poor rotamers (%)	0.3	0.2	0.5	0.1	0	0.1	0.1	0.1	0.1	0.2
Ramachandran plot										
Favored (%)	97.60	96.19	96.55	97.35	97.04	97.18	97.45	97.75	97.41	97.15
Allowed (%)	2.4	3.81	3.45	2.65	2.96	2.82	2.55	2.25	2.59	2.85
Disallowed (%)	0	0	0	0	0	0	0	0	0	0

Refinement statistics and validation information for each model.

**Table 2:** Cryo-EM data collection, refinement, and validation statistics for the 30 min datasets

Name	S4	I4	B5	I5	I6	I7	B6	B7	B8
State	Straight	Interm	Bent	Interm/Lis1	Interm/1xLis1	Interm/2xLis1	Bent/Lis1	Bent/1xLis1	Bent/2xLis1
Condition (Lis1)	n/a	n/a	n/a	Lis1	Lis1	Lis1	Lis1	Lis1	Lis1
Time point (min)	30	30	30	30	30	30	30	30	30
EMD	EMD-46953	EMD-46954	EMD-46959	EMD-46974	EMD-46972	EMD-46975	EMD-47033	EMD-47026	EMD-47032
PDB	9DKD	9DKE	9DKJ	9DLD	9DKX	9DLE	9DNB	9DN5	9DN7
**Data collection and processing**									
Magnification	150,000								
Voltage (kV)	200								
Electron exposure (e^–^/Å^2^)	54			54					
Defocus range (μm)	0.75–2.8								
Pixel size (Å)	0.889								
Symmetry imposed	*C*_1_								
Images (no.)	7,227			6,670					
Initial particles (no.)	1,047,074			744,012					
Final particles (no.)	119,768	94,448	122,388	106,139	59,650	40,384	67,155	35,055	28,077
Map resolution (Å) (FSC 0.143)	3.3	3.6	2.8	3.2	3.4	3.4	3	3.3	3.25
**Refinement**									
Initial model used (PDB code)	7MGM	7MGM	7MGM	7MGM	7MGM	7MGM	7MGM	7MGM	7MGM
Model resolution (Å) (FSC 0.5)	3.6	4.2	3.0	3.4	3.7	3.7	3.3	3.7	3.5
Map sharpening *B* factor (Å^2^)	82.3	74.3	71.2	77.2	70.1	56.5	57.1	55.1	51.8
Model composition									
Non-hydrogen atoms	17,901	17,457	18,075	22,996	20,808	22,893	22,963	21,000	23,772
Protein residues	2,313	2,291	2,291	3,017	2,646	2,985	3,020	2,679	3,021
Ligands	MG: 1, ADP :3, ATP: 1	MG: 1, ADP: 3, ATP: 1	MG: 2, ADP: 3, ATP: 1	MG: 3, ADP: 3, ATP: 1	MG: 3, ADP: 3, ATP: 1	MG: 1, ADP: 3, ATP: 1	MG: 1, ADP: 3, ATP: 1	MG: 1, ADP: 3, ATP: 1	MG: 1, ADP: 3, ATP: 1
*B* factors (Å^2^)									
Protein	67.01	123.03	66.14	96.14	106.31	108.74	69.30	99.61	84.55
Ligand	30.25	86.62	39.03	55.26	69.26	71.52	32.12	69.54	62.99
R.m.s. deviations									
Bond lengths (Å)	0.003	0.003	0.003	0.003	0.003	0.003	0.002	0.003	0.003
Bond angles (°)	0.655	0.715	0.628	0.597	0.621	0.600	0.521	0.623	0.579
**Validation**									
MolProbity score	1.50	1.43	1.19	1.48	1.46	1.49	1.37	1.35	1.52
Clashscore	7.00	6.2	4.06	6.19	5.41	5.76	6.71	6.43	6.17
Poor rotamers (%)	0.1	0.1	0.1	0.1	0.1	0.2	0	0	0
Ramachandran plot									
Favored (%)	97.44	97.58	98.01	97.27	97.05	97.01	98.25	98.49	96.91
Allowed (%)	2.56	2.42	1.99	2.73	2.95	2.99	1.75	1.51	3.09
Disallowed (%)	0	0	0	0	0	0	0	0	0

Refinement statistics and validation information for each model.

**Extended Data Fig. 5:**
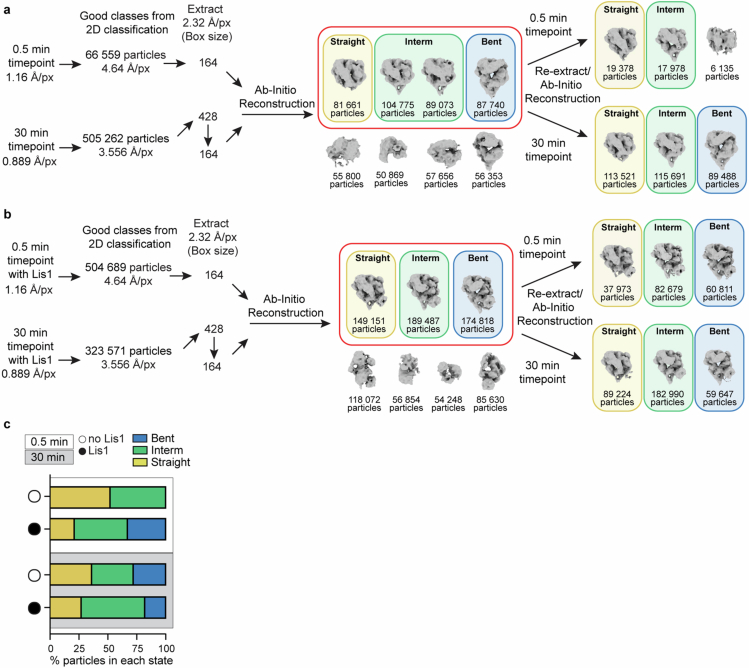
Cryo-EM data processing of the combined datasets. **a**. and **b**. Cryo-EM data processing workflow for the combined datasets. **c**. Relative abundance of particles belonging to different states obtained from particle distributions in cryo-EM datasets in the absence (open circle) or presence (black circle) of Lis1 at two different time points (0.5-min – white background and 30min – gray background). Source data

To explore how Lis1 binding affects dynein’s conformational landscape, we next examined the number of total particles that belong to each conformation (bent, blue; intermediate, green; and straight, yellow) in each dataset collected at the two time points and in the presence or absence of Lis1 (Fig. 1g). Although the individual conformations in each sample (dynein or dynein + Lis1) at different time points were the same, their distribution changed. Our analysis revealed that more conformations are present in the dynein-alone sample when the incubation is extended from 0.5 min to 30 min (Fig. 1g,h). However, the addition of Lis1 increases the number of conformations in dynein samples at the shorter time point (0.5 min) (Fig. 1g,h). To ensure that these differences were not due to variations in dataset size, we repeated the analysis by combining data belonging to the same experimental condition (+Lis1 or –Lis1) but were collected at different time points (Extended Data Fig. 5a,b). Because these datasets were collected using different microscopes, we processed them together only up to the generation of the initial three-dimensional (3D) reconstructed models, without any prior structural information. We mapped the particles from each model to their corresponding datasets and conformational subgroups (bent, intermediate, and straight linker). This analysis revealed an increase in the number of conformational states observed in the presence of Lis1 at the earlier time point, comparable to what we observed when each dataset was processed separately (Extended Data Fig. 5c). This suggests that Lis1 might facilitate dynein’s transition between different conformations, allowing dynein to sample more states. Next, we will discuss the different dynein conformations we observed in the absence and presence of Lis1, followed by how these conformations might explain the effects of Lis1 on dynein’s mechanochemistry.

### Dynein’s conformational landscape during ATP hydrolysis

**Fig. 2:**
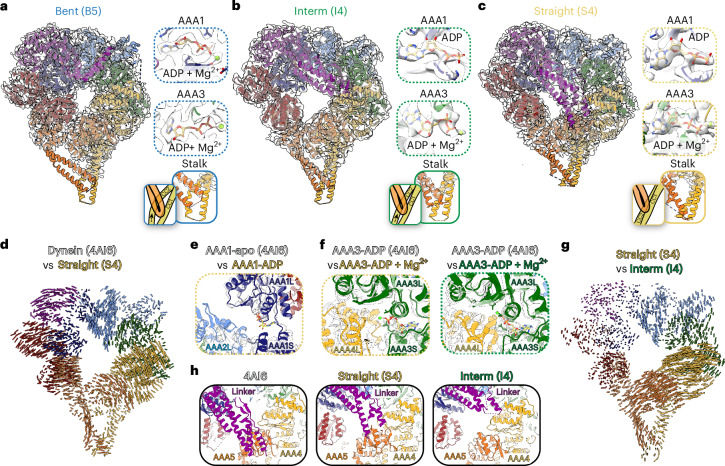
Conformational landscape of dynein during ATP hydrolysis. **a–c**, Models of dynein for the bent (B5) (**a**), intermediate (I4) (**b**), and straight (S4) (**c**) conformations from the 30 min dataset fit into their corresponding cryo-EM density maps. Each structural element is colored according to the dynein schematic in Fig. 1a. To the right of each fitted model are close ups of the nucleotide-binding pockets for AAA1 and AAA3, and of the predicted stalk conformations. **d**, A map of pairwise alpha-carbon distances between the straight linker model (S4) and an X-ray structure of *S. cerevisiae* dynein in apo-AAA1 (PDB: 4AI6), with the models aligned relative to their AAA1 modules. The length of each vector is proportional to the interatomic distance. Residues 1361–1772 of the linker were removed for clarity. **e**, Comparison of AAA1 nucleotide-binding pockets for straight linker dynein (S4) bound to ADP with *S. cerevisiae* in apo-AAA1 (PDB: 4AI6). The models were aligned based on residues 1797–1894 in AAA1. **f**, Comparison of AAA3 nucleotide-binding pockets for straight linker dynein (S4, left panel, yellow outline) and intermediate linker dynein (I4, right panel, green outline) with *S. cerevisiae* (PDB: 4AI6). The models were aligned based on residues 2377–2445 in AAA3. **g**, Map of pairwise alpha-carbon distances between the straight linker (S4) and the intermediate linker (I4) models. The length of each vector is proportional to the interatomic distance. The models were aligned relative to their AAA1 modules. Residues 1361–1772 of the linker were removed for clarity. **h**, View of linker docking in PDB 4AI6 (left), straight linker conformation (S4, middle), and intermediate linker conformation (I4, right).

**Extended Data Fig. 6:**
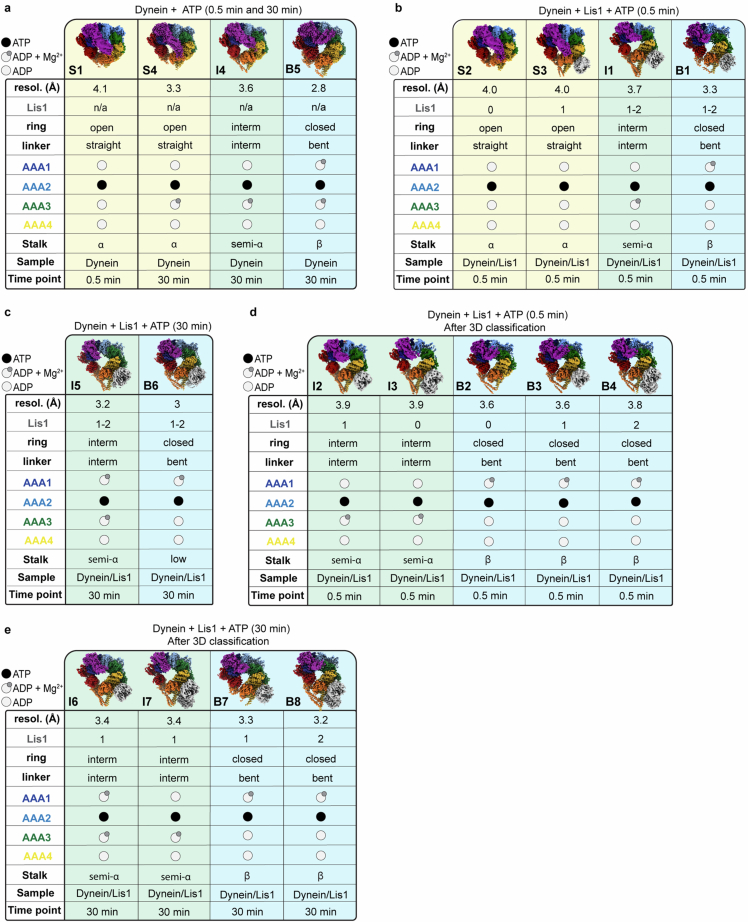
Summary of the identified structures and their properties. Summary of the cryo-EM volumes determined in this work, with their properties, for the indicated datasets. Stalk represents the identified stalk conformations^51^. S – straight linker conformation, yellow background; I – intermediate linker conformation, green background; and B – bent linker conformation, blue background. **a.** Dynein + ATP, 0.5 min and 30 min datasets. **b.** Dynein + ATP + Lis1, 0.5 min dataset. **c.** Dynein + Lis1 + ATP, 30 min dataset. **d.** Dynein + Lis1 + ATP, 0.5 min dataset after 3D classifications. **e.** Dynein + Lis1 + ATP, 30 min dataset after 3D classifications.

To understand how ATP hydrolysis affects dynein structurally, we built models of different high-resolution dynein conformations identified in our datasets. We mapped nucleotides to their binding pockets, as all our structures showed nucleotide densities in the AAA modules (Fig. 2a–c and Extended Data Figs. 4a–c and 6a). In each linker state (bent, intermediate, and straight) dynein is bound to ADP in AAA1 and AAA3, although in some states there is also density for Mg^2+^, indicating a tighter binding state because Mg^2+^ release weakens ADP binding^49,50^. Although our maps have only partial densities in the stalk region, the ring opening and the stalk’s CC1 and CC2 positions in dynein with a straight linker is consistent with a strong microtubule-binding state, as indicated by the lack of bulging of CC2 in the stalk region characteristic of the weak microtubule-binding state (Fig. 2a,c)^51^. The intermediate linker dynein has partial density that fits a model in which the CC2 has a slight bulge in the stalk and is consistent with a semi-bent, also called semi-α state that could represent an intermediate microtubule binding state (Fig. 2b)^19,52,53^. In agreement with having occupied nucleotide binding pockets, the ring of the straight linker dynein is more closed than in the crystal structure of apo-dynein (PDB: 4AI6, Fig. 2d). We see a partial closing of the AAA1 binding pocket, indicative of a post-hydrolysis ADP-bound state (Fig. 2e), and ADP and Mg^2+^ in AAA3 in both straight and intermediate dynein as opposed to ADP without Mg^2+^ in the bent linker dynein (Fig. 2a) or crystal structure (PDB: 4AI6, Fig. 2f)^16^.

The intermediate linker dynein states identified in our datasets have similar conformations to the ones identified in a recent analysis of full length human dynein^54^. In our dataset, the comparison of the straight and intermediate linker dynein shows movement in the AAA domains, even though the nucleotide states are the same in these structures (Fig. 2b,c,g). The linker docks at AAA5 in the apo (PDB: 4AI6) and straight linker dynein, but it docks at AAA4 in the intermediate linker dynein (Fig. 2h). These differences in the ring and linker without corresponding changes in the nucleotide state suggest some flexibility in dynein that is independent of ATP hydrolysis and is consistent with previous FRET analysis and recent human dynein structures^12,54^.

### The effect of Lis1 on dynein’s conformational landscape

**Fig. 3:**
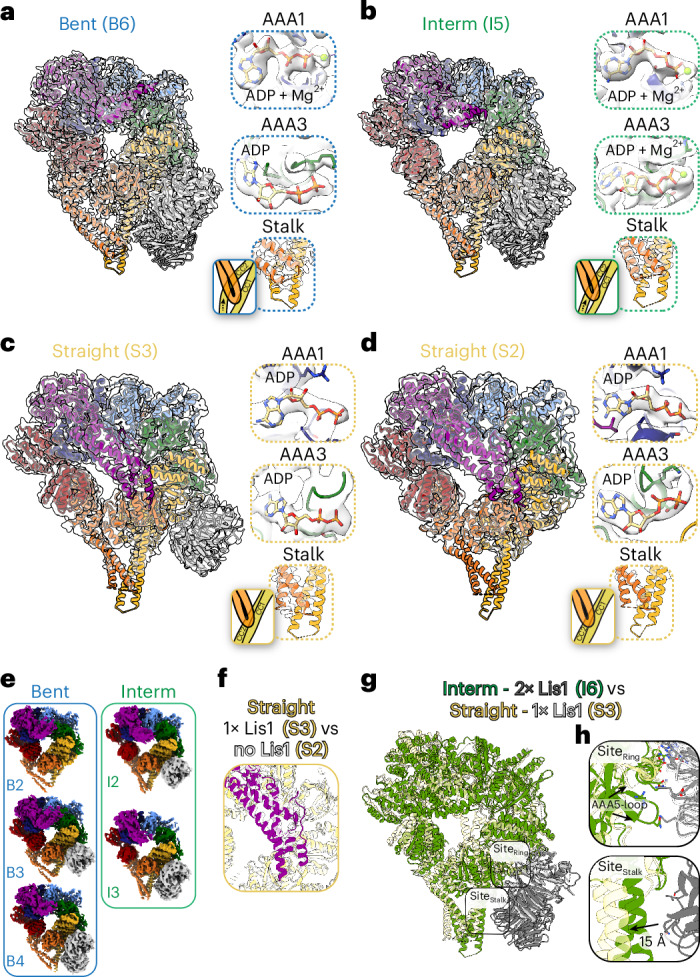
Lis1 binding to dynein expands dynein’s conformational landscape. **a–d**, Models of dynein bound to Lis1 for the bent (B6) (**a**) and intermediate (I5) (**b**) dynein from the same dataset (dynein with Lis1 incubated for 30 min with ATP), and straight with Lis1 bound (S3) (**c**) and straight with no Lis1 bound (S2) (**d**) dynein from the same dataset (dynein with Lis1 incubated for 0.5 min with ATP), fit into their corresponding cryo-EM density maps. Each structural element is colored according to the dynein schematic in Fig. 1a. To the right of each fitted model are views of the nucleotide binding pockets for AAA1 and AAA3 and of the stalk conformations. **e**, Additional cryo-EM maps obtained after heterogeneity analysis of bent (blue box) and intermediate (green box) dynein conformations from the dynein with Lis1 incubated for 0.5 min with ATP dataset after 3D classification show one or two Lis1 bound to dynein. **f**, Comparison of linker (magenta) in straight linker dynein (S3, yellow) bound to Lis1 with linker (white) in straight linker dynein (S2, white) with no Lis1 bound. The models were aligned on the basis of the position of AAA1. **g**, Comparison of models built for intermediate state dynein (I6, green) bound to two Lis1 β-propellers (gray) with straight linker dynein (S3, yellow) bound to one Lis1 β-propeller (white). The two Lis1-binding sites are highlighted. The models were aligned on the basis of the position of Lis1 bound to site_ring_. **h**, The dynein and Lis1 binding sites from **g**.

To understand how Lis1 affects dynein’s mechanochemistry, we next analyzed the datasets in which dynein was pre-incubated with Lis1 before the addition of ATP. These datasets exhibited a high level of heterogeneity (Supplemental Video 1) with the bent and intermediate linker dynein states in the 0.5 min and 30 min conditions (Fig. 3a,b and Extended Data Figs. 6b,c) while the straight linker states where detected only in the 0.5 min condition (Figs. 1g,h and 3c,d and Extended Data Fig. 6b). In addition, the nucleotide occupancies and stalk conformations in these different dynein states are similar to those seen in the absence of Lis1 (Figs. 2a–c and 3a–d and Extended Data Figs. 4 and 6).

**Extended Data Fig. 7:**
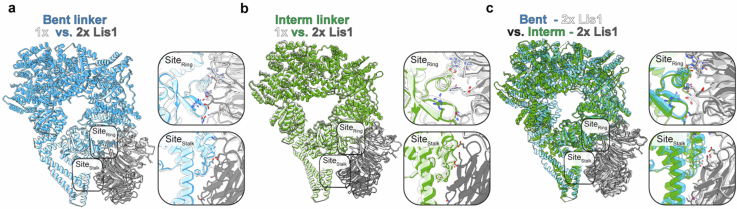
Specifics of Lis1 interaction with dynein. **a**. Comparison of models build for bent linker dynein bound to 2x Lis1 β-propellers (B8, blue, Lis1 – gray) with bent linker dynein bound to 1x Lis1 β-propellers (B7, white, Lis1 – white). The two Lis1 binding sites are highlighted and shown to the right. **b**. Comparison of models build for intermediate linker dynein bound to 2x Lis1 β-propellers (I7, green, Lis1 – gray) with intermediate linker dynein bound to 1x Lis1 β-propellers (I6, white, Lis1 – white). The two Lis1 binding sites are highlighted and shown to the right. **c**. Comparison of models build for bent linker dynein bound to 2x Lis1 β-propellers (B8, blue, Lis1 – white) with intermediate linker dynein bound to 2x Lis1 β-propellers (I7, green, Lis1 – white). The two Lis1 binding sites are highlighted and shown to the right.

Because some of our maps showed partial density for the Lis1 β-propellers, we performed 3D classifications without alignment in Relion to further resolve this compositional heterogeneity (Extended Data Figs. 2 and 3). This analysis distinguished states with no, one, or two Lis1 β-propellers bound to dynein (Fig. 3e and Extended Data Figs. 2–4 and 6d,e). The nucleotide occupancies in these new maps correspond to the nucleotides observed in pre-3D classification maps (Fig. 3a–d and Extended Data Figs. 4 and 6). In addition, the interaction sites between Lis1 and dynein in our maps are those previously characterized and are largely the same in the bent and intermediate dynein states (Extended Data Fig. 7a–c).

### Dynein linker position controls binding to a second Lis1

Previous low-resolution data showed that dynein in a strong microtubule-binding state binds one Lis1 β-propeller at site_ring_^43^. However, that structure was determined in a straight linker state, highlighting the linker position as a crucial factor in Lis1 binding to dynein. Our straight linker map also had one Lis1 β-propeller bound (Fig. 3c). Aligning straight linker dynein with or without Lis1 by AAA1 shows that the linker shifts slightly to accommodate Lis1 binding (Fig. 3f). We also saw clear density for the second Lis1 β-propeller in the intermediate state dynein, even though the nucleotide densities in this state are consistent with the ones in the straight linker dynein (Fig. 3b and Extended Data Figs. 4b and 6). Comparison of this state (I6, 3.4-Å resolution) with our straight linker dynein (I3, 4-Å resolution) bound to one Lis1 β-propeller showed that the site_ring_ interactions are conserved while site_stalk_ is shifted away from Lis1 as dynein goes from the intermediate to the straight linker state (~15 Å), making it inaccessible for binding by the second Lis1 β-propeller (Fig. 3g,h). Binding of Lis1 at the second site is unlikely to happen without Lis1 bound at site_ring_. This is consistent with previous lower-resolution structures suggesting that the straight linker dynein conformation is incompatible with the second Lis1 β-propeller binding^35,43,44^.

In our straight linker dynein bound to one Lis1 at site_ring_, the density for the AAA5 loop is missing, but it is present in the intermediate or bent linker structures bound to one or two Lis1 (Fig. 3h and Extended Data Fig. 7a). Notably, substitutions in this loop (at N3475 and N3476), or of the corresponding residues D253 and H254 in Lis1, disrupts dynein’s interaction with Lis1 and leads to binucleate phenotypes in yeast, suggesting that this interaction might only take place when dynein transitions from straight to intermediate or bent linker states^35^.

### Lis1 increases dynein’s basal ATP hydrolysis rate

**Fig. 4:**
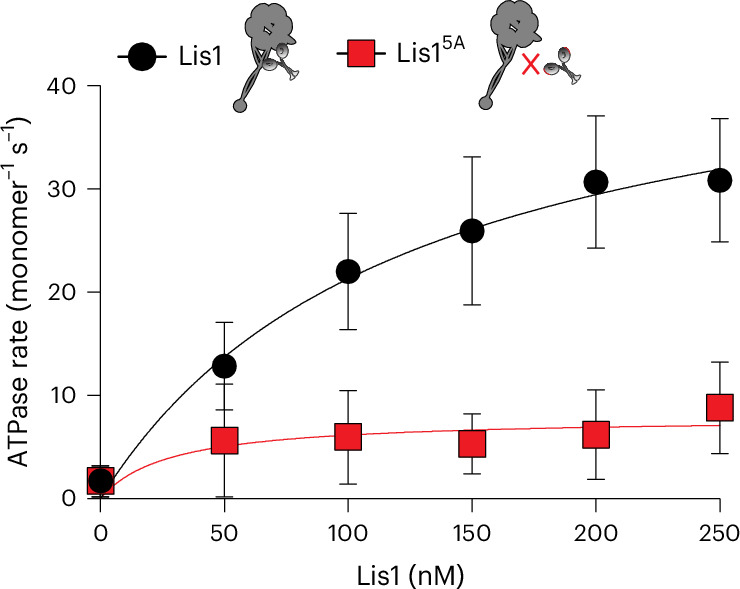
Lis1 increases dynein’s basal ATPase activity. ATPase activity of dynein in the presence of increasing concentrations of wild-type Lis1 (black dots) or a Lis1 mutant that does not bind to dynein (Lis1^5A^, red squares). Mean values (± s.d.) from three independent experiments are shown. Each experiment was performed in duplicate. Fitted values (± standard error of the fit); *k*_basal_ (basal rate) = 0.837 ± 0.08 s^–1^, *k*_cat_[Lis1] (turnover rate) = 25.75 ± 2.4 s^–1^. Source data

Our analysis showed that dynein sampled more conformations at the shorter time point in the presence of Lis1 (Fig. 1g,h and Extended Data Fig. 5c). We hypothesized that Lis1 could accomplish this by increasing dynein’s basal ATP hydrolysis rate. Several studies have shown that Lis1 does not affect dynein’s microtubule-stimulated ATPase activity^22,44,46^. However, the contribution of Lis1 to dynein’s basal ATPase hydrolysis rate (in the absence of microtubules) has not been fully established^22,44^. To test our hypothesis, we incubated the same dynein monomer (amino acids 1352–4092) as the one used in cryo-EM studies with increasing concentrations of Lis1 and measured dynein’s basal ATP hydrolysis rate. We observed a Lis1-concentration-dependent increase in the ATP hydrolysis rate (Fig. 4). Of note, binding of Lis1 to dynein is required for this effect, as the rate did not increase in the presence of a Lis1-R275A R301A R378A W419A K437A (Lis1^5A^), which does not bind to dynein (Fig. 4)^36,43,44^. This observation supports our hypothesis that Lis1 binding allows dynein to sample more conformations by increasing dynein’s basal ATP hydrolysis rate.

### Dynein activation is driven by its mechanochemical cycle

To understand how Lis1 increases dynein’s ATPase rate, we further analyzed the interactions between the linker and the dynein motor domain, as these interactions have been previously shown to be important for dynein’s mechanochemistry^12,44,55^. We reasoned that the linker conformation in the different dynein states could explain how Lis1 regulates dynein’s mechanochemistry given the changes in linker position associated with ATP hydrolysis and Lis1 binding.

**Fig. 5:**
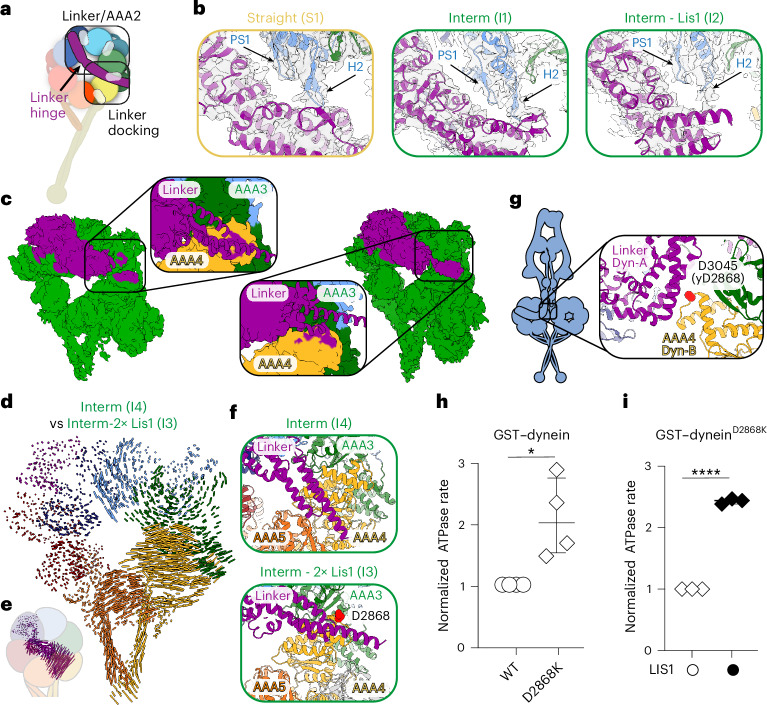
Conformational changes and Lis1 binding regulate dynein’s basal ATP-hydrolysis rate. **a**, Schematic of the two important interaction regions between dynein’s linker and motor domain. **b**, The proximity of the P1 sensor (PS1) and H2 insert loops in AAA2 (blue) to the linker (magenta) hinge region in the indicated models. **c**, Density maps and close-ups for subsets of particles selected from cryoDRGN analysis that showed the most extensive linker density. Close-ups show predicted linker (magenta) docking sites on AAA4 (yellow) after fitting an extended linker model in the density maps. **d**, Map of pairwise alpha-carbon distances between the intermediate linker model and the intermediate linker model bound to two Lis1 β-propellers. The models were aligned by their AAA1 modules. Residues 1361–1772 of the linker were removed for clarity. **e**, Residues 1352–1772 of the linker from the map of pairwise alpha-carbon distances between the intermediate linker model and the intermediate linker model bound to two Lis1 β-propellers in **d**. **f**, View of linker docking in intermediate linker dynein bound to Lis1 β-propellers (top) and intermediate linker dynein (bottom). Residue D2686 is highlighted in red. **g**. View of the location of residue D3045 (yeast D2868) in human dynein Phi particle (PDB: 5NVU). **h**, Normalized ATPase activity (median ± interquartile range) of GST–dynein and GST–dynein^D2868K^. Unpaired *t*-test with two-tailed *P* value; *P = 0.0145. Data from four independent experiments. Each experiment was performed in duplicate. **i**, Normalized ATPase activity (median ± interquartile range) of GST–dynein^D2868K^ in the absence (white circles) or presence (black circles) of Lis1. Unpaired *t*-test with two-tailed *P*-value. ****P < 0.0001. Data are from three independent experiments. Each experiment was performed in duplicate. Source data

Two models for how swinging of the linker is regulated during ATP hydrolysis have been proposed^12,55,56^. In the linker/AAA2 model, loops in AAA2 (pre-sensory I (PS1) and helix 2 (H2)) rearrange during ATP hydrolysis in AAA1, leading to an interaction with the linker hinge and subsequent bending and swinging of the linker (Fig. 5a). Consistent with this, deleting the H2 region in *Dictyostelium* dynein reduces dynein’s basal and microtubule-stimulated ATPase activity^12^. In the straight and intermediate dynein state, when Lis1 is not bound to dynein, the linker interacts with the AAA2 H2 loop, but these contact sites are less resolved when Lis1 is bound, suggesting reduced engagement in this region (Fig. 5b). This could mean that when Lis1 binds to dynein, the need for this interaction is bypassed owing to the Lis1-driven increased flexibility in dynein.

In the linker docking model, ATPase-induced changes in AAA1 propagate to the linker docking interface on the motor domain to induce linker swing (Fig. 5a). Because our models lacked density for the linker docking sites when Lis1 was bound to dynein in the intermediate state, we performed heterogeneity analysis of all particles that belong to the intermediate state from the dataset collected in the presence of Lis1 and at the 0.5-min time point (Extended Data Figs. 2b and 4b). In addition to separating the populations that have one or two bound Lis1 β-propellers, we observed other conformational changes that correspond to linker movement (Extended Data Fig. 2a). We then picked particles from the states with the most linker density, regardless of whether they had one or two Lis1 β-propellers bound. Although this analysis did not improve the resolution of our maps, it did allow us to extend the modeling of the linker in the region where it could potentially dock on dynein. For each density map generated in this analysis, the linker comes close to AAA4 or the interface between AAA3 and AAA4 (Fig. 5c and Extended Data Fig. 2c).

We next compared these linker docking interfaces with intermediate-state dynein observed in the absence of Lis1. The presence of Lis1 led to conformational changes in AAA1–AAA5 (Fig. 5d). The linker is also shifted, with the docking site much closer to the interface between AAA4 and AAA5 when Lis1 is not present (Fig. 5e,f). In Lis1-bound dynein, the linker is positioned near D2868 (corresponding to D3045 in human dynein), which makes important contacts with the linker of the opposing dynein in the Phi particle (Fig. 5f,g)^5^. A charge-reversing mutation in this residue disrupts Phi particle formation and nearly eliminates the requirement for Lis1 in yeast^5,41^. Given that Phi traps dynein in a single conformation, and that conformational changes, such as opening and closing of the ring and linker swing, are required for dynein to go through its mechanochemical cycle, we reasoned that the Phi conformation inhibits dynein’s mechanochemistry, in addition to preventing it from binding to microtubules. This suggests that disrupting Phi particle formation would increase dynein’s basal ATPase hydrolysis rate. To test this, we used GST–dynein because the Phi-disrupting D2868K substitution in this construct does not affect dynein’s motile properties or Lis1 binding^41^. The ATPase hydrolysis rate of GST–dynein-D2868K was almost double that of the wild-type construct, suggesting that the Phi conformation inhibits dynein’s ability to hydrolyze ATP (Fig. 5h). The addition of Lis1 continued to increase dynein’s ATPase rate, implying that other interactions are likely important to this process (Fig. 5i).

### Lis1 might be facilitating nucleotide release from AAA3

**Fig. 6:**
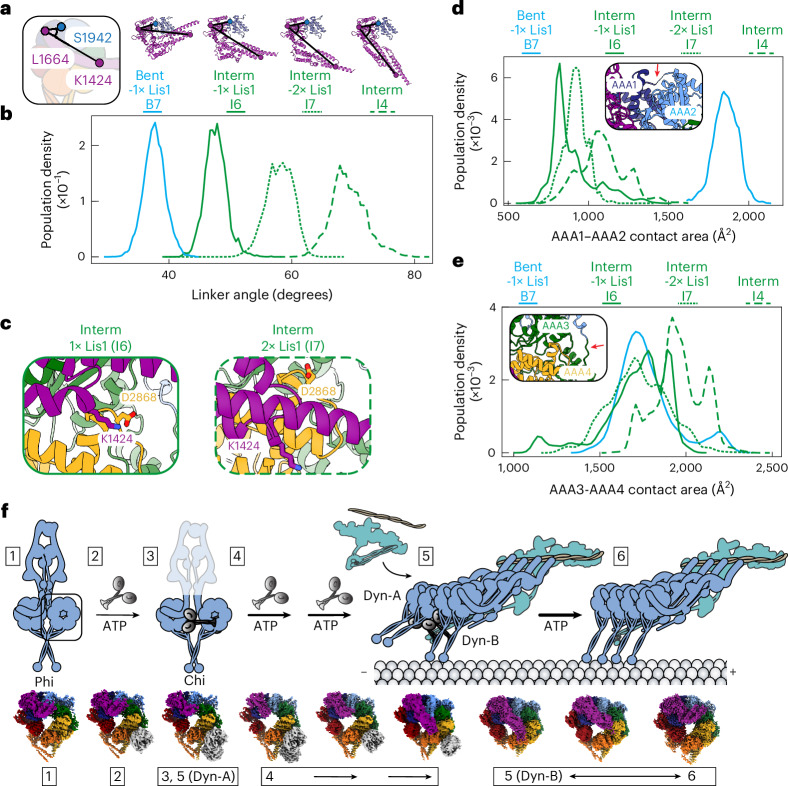
Molecular dynamics support changes in dynein conformations due to Lis1 binding. **a**, The linker rotation angles defined by the Cα atoms of residues located in AAA2 (S1942, corner), linker–corner (L1664–S1924, edge 1), and linker–corner (L1664–K1424, edge 2) for the four systems used in the molecular dynamic simulations: bent linker with one Lis1 β-propellers bound (B7, solid blue line), intermediate linker with one Lis1 β-propeller bound (I6, solid green line), intermediate linker with two Lis1 β-propellers bound (I7, dotted green line), and intermediate linker (I4, large dot green line). **b**, The population density for the linker rotation angles for the four simulation systems. **c**, The predicted salt bridge between D2868 in AAA3 and K1424 in the linker for intermediate linker with one Lis1 β-propeller bound (I6, top) and intermediate linker with two Lis1 β-propellers bound (I7, bottom). **d**, The distribution of the contact area surface between AAA1 and AAA2 (insert, red arrow points to the interface) in the simulations. **e**, Distribution of contact area surface between AAA3 and AAA4 domains (insert, red arrow points to the interface) in the simulations. **f**, Model of dynein activation and the proposed placement of the identified structures in the model. (1) Dynein in the Phi particle, (2) the initial step of Chi particle formation, (3) the Chi particle, (4) the increase in dynein’s basal ATP hydrolysis rate, during which dynein likely samples intermediate states, (5) the full dynein complex assembles with dynactin and an activating adapter that binds to microtubules and contains one set of dynein dimers bound to Lis1 (Dyn-A) and another set of dynein dimers without Lis1 (Dyn-B), and (6) Lis1 dissociates from the active dynein complex. The thickness of the arrows in the top panel indicates relative ATP hydrolysis rates. The double-headed arrow indicates dynein’s movement on microtubules, during which dynein samples multiple conformational states. Source data

To gain a deeper understanding of Lis1’s effects on dynein’s mechanochemistry, we turned to all-atom molecular dynamic (MD) simulations. We first asked how the presence of Lis1 influences dynein’s linker bending, given the importance of linker position in the mechanochemical cycle (Fig. 5a)^12,55^. To do this, we first defined the linker rotation angle between residues S1942 (AAA1), L1664 (linker), and K1424 (extended part of the linker) in our highest-resolution dynein models obtained in this work: bent-1× Lis1 (Fig. 3e), interm-1× Lis1 (Fig. 5d), interm-2× Lis1 (Fig. 5d), and interm dynein (Fig. 2b), and determined from heterogeneity analysis, in which we modelled the same number of residues for the linker region (Methods and Fig. 6a). For each state, we launched multiple Gaussian Accelerated Molecular Dynamics (MD) simulations (Methods), which can enhance the conformational sampling of molecular machines, and measured the population density across different linker angles. Our analysis shows that the linker rotation is more constrained when Lis1 is bound to dynein in the intermediate state (Fig. 6b). This is likely caused by the steric hindrance from Lis1 binding to dynein.

We next evaluated whether the residue D2868, crucial for Phi particle formation, could be making contacts with the linker when Lis1 is bound to dynein. We found that D2868 forms a salt bridge with K1424 (linker), which could stabilize the intermediate linker position when Lis1 is bound to dynein in this state (Fig. 6c). This salt bridge is relatively stable in the simulation with the intermediate dynein bound to one Lis1 β-propeller (Fig. 6c), whereas a disengaged configuration is preferred in the intermediate dynein bound to two β-propellers (Fig. 6c).

Last, we asked whether the presence of Lis1 could affect the nucleotide-binding pockets in AAA1 and AAA3 in our simulations. The analysis of the contact area between AAA1 and AAA2 revealed that the bent linker enables a tighter interface than that in the intermediate linker state, presumably allowing this site to become more hydrolysis competent (Fig. 6d). This is consistent with closing of the AAA1 interface in the bent conformation seen for *Dictyostelium* dynein in the pre-power stroke state primed for ATP hydrolysis^51^. We next investigated the AAA3 and AAA4 contact area and showed that Lis1 binding loosens the AAA3 and AAA4 interface, potentially favoring ADP release from the AAA3 binding pocket (Fig. 6e). Notably, binding of the second Lis1 β-propeller further loosens the interface, as illustrated by comparing the population distributions of interm-2× Lis1 versus interm-1× Lis1 systems (Fig. 6e). This analysis suggests that Lis1 might increase dynein’s basal ATP hydrolysis rate by facilitating nucleotide release from AAA3.

## Discussion

Our work showed how heterogeneity mining of cryo-EM data combined with time-resolved approaches can identify a conformational landscape for dynein during its mechanochemical cycle and how this landscape is altered by Lis1 binding. The conformations identified in our study are in line with a recent similar analysis of full-length human dynein^54^. However, our work goes further, defining the contributions of Lis1 to dynein’s mechanochemistry. Previous attempts to capture the different steps of dynein’s mechanochemical cycle using X-ray crystallography were not fully comprehensive^15^. Combining heterogeneity mining with time-resolved cryo-EM provides a unique advantage in visualizing how ATP hydrolysis drives changes in dynein’s conformational landscape over time.

We identified multiple conformations of dynein during ATP hydrolysis and determined the nucleotide state of each AAA module. Our various dynein and dynein–Lis1 structures can be mapped onto the dynein activation pathway, starting with the bent linker dynein without Lis1. This conformation is consistent with individual dynein protomers in Phi (Fig. 6f (1)) and conformations that dynein samples while in the assembled complex moving along microtubules (Fig. 6f (6)). The bent linker dynein with one Lis1 β-propeller bound at site_ring_ likely represents an early step in the formation of the Chi particle (Fig. 6f (2)), followed by binding of the second Lis1 β-propeller at site_stalk_ and full assembly of Chi (Fig. 6f (3)). We also identified a small population of particles in our datasets that corresponded to Chi dynein, containing two dyneins and two Lis1 dimers (published previously in ref. ^39^). Thus, our analysis is consistent with the idea that Lis1 binding to dynein and Chi formation is an early step in dynein activation.

We propose a model in which Lis1 binding to dynein initially disrupts the autoinhibited Phi conformation by forming the Chi particle (Fig. 6f (3))^39^. Once bound, Lis1 allows dynein to go through its mechanochemical cycle, which is seen as an increase in dynein’s basal ATP hydrolysis rate (Fig. 6f (4)). This likely both disrupts Chi and allows dynein to adopt intermediate conformations (Fig. 6f (4)) compatible with complex assembly with dynactin and an activating adapter (Fig. 6f (5)). Interestingly, we did not observe intermediate linker dynein by itself in any of our datasets collected in the presence of Lis1, although we could solve structures of straight and bent linker dynein with and without Lis1 bound from those same datasets (Fig. 1g and Extended Data Fig. 4a,b). In addition, at the 30 min time point and in the presence of Lis1, the intermediate state dynein was the dominant one (Fig. 1h and Extended Data Fig. 5c). Although we cannot rule out that sample preparation somehow led to these distributions, this could mean that binding of Lis1 to dynein is particularly favorable in the intermediate state. Once the initial complex assembles, and the first dynein dimer (Fig. 6f (5, Dyn-B)) binds to microtubules, Lis1 bound to the second dynein dimer (Fig. 6f (5, Dyn-A)) might dissociate to allow for dynein’s movement on the microtubules, during which dynein samples multiple conformations (Fig. 6f (6)). It is also possible that Lis1 remains bound to active dynein given a recent study showing colocalization of moving dynein and Lis1 in neuronal cells^57^. The dynein conformations we observed in the absence of Lis1 likely represent the different steps of dynein’s ATP-hydrolysis-driven movement on microtubules (Fig. 6f (6)). Further structural work with full-length dynein, dynactin, an activating adapter, and Lis1 in the presence of ATP will be required to fully map how dynein is activated.

We showed direct evidence that Lis1 stimulates dynein’s basal ATP hydrolysis rate and suggest that this basal activity is inhibited when dynein is in the Phi conformation. In accordance with this, the D2868K substitution in dynein that prevents Phi formation increases dynein’s basal ATPase rate. However, Lis1 stimulates dynein’s ATPase activity further after the Phi particle is disrupted. This suggests a two-step regulation mechanism. In the first step, Lis1 binds to dynein to open the Phi particle, which results in a higher rate of ATP hydrolysis as the conformational constraints imposed by Phi are released. Once Phi has been disrupted, Lis1 further increases dynein’s ATPase rate by acting on the released motor, possibly by facilitating the release of ADP from AAA3. This could be driven by the decreased communication between the linker and dynein’s motor domains, as suggested by our MD simulations. There, Lis1 binding to dynein in the intermediate state seemed to be influencing the ability of the linker to communicate with AAA4 (D2868) and the H2 loops in AAA2 (Fig. 5b). Further structural and mechanistic work will be required to fully decipher this mechanism.

## Methods

### Yeast strains construction

The *S. cerevisiae* strains used in this study are listed in Extended Data Table 1. The endogenous genomic copies of *PAC1* (encoding Lis1) or *DYN1* (encoding dynein) were deleted using PCR-based methods, as previously described^58^. A point mutation to generate dynein-D2868K was introduced using QuikChange site-directed mutagenesis (Agilent) with the primers 5′-CTTTAGGTCTTTTATTGAAGACAGAACAAGAACTG-3′ and 5′-CAGTTCTTGTTCTGTCTTCAATAAAAGACCTAAAG-3′ and verified by DNA sequencing. The mutant fragment was re-inserted into the kl*URA3* strains using the lithium acetate method^59^ to reintroduce the mutated *DYN1* gene. Positive clones (kl*URA3*) were selected in the presence of 5-fluorootic acid, screened by colony PCR, and verified by DNA sequencing.

### Protein expression and purification

Protein purification steps were done at 4 °C unless otherwise indicated. *S. cerevisiae* dynein constructs were purified from *S. cerevisiae* using a ZZ tag, as previously described^60^. In brief, liquid-nitrogen-frozen yeast cell pellets were lysed by grinding in a chilled coffee grinder and resuspended in dynein-lysis buffer supplemented with 0.5 mM Pefabloc, 0.05% Triton X-100, and cOmplete EDTA-free protease inhibitor cocktail tablet (Roche). The lysate was clarified by centrifugation at 264,900 × *g* for 1 h. The clarified supernatant was incubated with IgG Sepharose beads (GE Healthcare Life Sciences) for 1 h. The beads were transferred to a gravity flow column, washed with dynein-lysis buffer supplemented with 250 mM potassium chloride, 0.5 mM Pefabloc, and 0.1% Triton X-100, and with TEV buffer (10 mM Tris–HCl (pH 8.0), 150 mM potassium chloride, 10% glycerol, and 1 mM DTT). Dynein was cleaved from IgG beads through incubation with 0.15 mg ml^–1^ TEV protease (purified in house) overnight at 4 °C. Cleaved dynein was concentrated using a 100 K MWCO concentrator (EMD Millipore), filtered by centrifugation with a Ultrafree-MC VV filter (EMD Millipore) in a tabletop centrifuge, and used fresh for cryo-EM sample preparation or snap-frozen in liquid nitrogen.

Yeast Lis1 was purified from *S. cerevisiae* using 8×His and ZZ tags, as previously described^22^. In brief, liquid-nitrogen frozen pellets were ground in a prechilled coffee grinder, resuspended in buffer A (50 mM potassium phosphate (pH 8.0), 150 mM potassium acetate, 150 mM sodium chloride, 2 mM magnesium acetate, 5 mM β-mercaptoethanol, 10% glycerol, 0.2% Triton X-100, 0.5 mM Pefabloc) supplemented with 10 mM imidazole (pH 8.0) and cOmplete EDTA-free protease inhibitor cocktail tablet, and spun at 118,300 × *g* for 1 h. The clarified supernatant was incubated with Ni-NTA agarose (QIAGEN) for 1 h. The Ni beads were transferred to a gravity column, washed with buffer A with 20 mM imidazole (pH 8.0), and eluted with buffer A with 250 mM imidazole (pH 8.0). The eluted protein was incubated with IgG Sepharose beads for 1 h. IgG beads were transferred to a gravity flow column, washed with buffer A with 20 mM imidazole (pH 8.0) and with modified TEV buffer (50 mM Tris–HCl (pH 8.0), 150 mM potassium acetate, 2 mM magnesium acetate, 1 mM EGTA, 10% glycerol, 1 mM DTT). Lis1 was cleaved from the IgG beads by the addition of 0.15 mg ml^–1^ TEV protease (purified in house) for 1 h at 16 °C. Cleaved proteins were filtered by centrifugation with Ultrafree-MC VV filter (EMD Millipore) in a tabletop centrifuge and flash frozen in liquid nitrogen.

### Electron microscopy sample preparation

To remove any residual nucleotide, purified fresh (not frozen) dynein was incubated with apyrase for 30 min (apyrase, 0.1 U ml^–1^, New England Biolabs) before gel filtration on a Superose 6 Increase column pre-equilibrated in buffer containing 50 mM Tris (pH 8), 150 mM KCl, 2 mM EDTA, and 1 mM DTT. Eluted protein was concentrated to about 5 µM and diluted 1:1 with Lis1 or TEV buffer and incubated on ice for 10 min before ATP addition (final concentrations, 2.5 µM dynein, 2.5 µM Lis1, and 1.25 mM ATP). Samples containing ATP were incubated for a total of 0.5 min or 30 min on ice (including blotting time and plunging) and quickly applied to plasma cleaned (Solarus, Gatan) UltrAuFoil Holey Gold R 1.2/1.3 µM3 grids (Quantifoil). A vitrobot (FEI) was used to blot away excess sample and plunge freeze the grids in liquid ethane. The Vitrobot chamber was maintained at 100% humidity and 4 °C during the process. Grids were stored in liquid nitrogen until imaging. The 0.5 min grids were made and analyzed first. On the basis of the observed conformational landscape in this condition, the 30 min time point was chosen for the longer time condition.

### Electron microscopy image collection

The 0.5-min time point grids were imaged using Talos Arctica operated at 200 kV and equipped with K2 Summit direct electron detector (Gatan). Automated data collection was performed using Leginon. A total of 1,849 videos over 3 sessions for the dynein + ATP, 0.5 min dataset and 12,370 movies over 4 sessions for the dynein + Lis1 + ATP, 0.5 min dataset at ×36,000 (1.16 Å per pixel) were collected. The dose was ~6.3 Å^–2^ s^–1^ with a total exposure time of 11 s divided into 200-ms frames, for a total of 40 frames. The defocus range was set to 0.8–2.4 µm.

The 30-min time point grids were imaged using an FEI Titan Krios (200 kV) equipped with a Flacon 4i detector and energy filter (<10 eV slit size) (Thermo Fisher). A total of 7,182 videos for the dynein + ATP, 30 min dataset and 6,670 videos for the dynein + Lis1 + ATP, 30 min dataset were collected using automated data acquisition (Thermo Fisher EPU) at ×130,000 (0.889 Å per pixel). The dose was ~5 Å^–2^ s^–1^ with a total exposure time of 10 s divided into 200-ms frames, for a total of 40 frames. The defocus range was set to 0.75–2.8 µm.

### Electron microscopy data processing

All videos were aligned in CryoSPRC live^61^ using MotionCor2 (ref. ^62^) of the dose-weighted frames. CTF was estimated in dose-weighted images using CTFFIND4 (ref. ^63^). Images with CTF fits above 5 Å were excluded from further processing. Particles were initially selected using blob finder in CryoSPRC, and these peaks were used for Topaz^64^ model training and final particle picking using Topaz. Particles were extracted and binned to 4.64 Å per pixel for the 0.5 min dataset and to 3.556-Å per pixel for the 30 min dataset. Multiple rounds of two-dimensional (2D) classification with a varying number of online-EM interactions (30–40) and a batch size per class between 200–400, depending on the number of particles in each classification, were first carried out in cryoSPARC to remove bad particles. Reconstructions were performed to generate initial models for each dataset with particles from all good 2D classes. Good particles and models were selected for multiple rounds of 3D refinement and local refinements until no further improvement was observed and models appeared to contain well-defined secondary structures. Further analysis of heterogeneity was performed using 3D classification without alignment in Relion-3 to find subsets of particles that were classified into models with well-defined secondary structures. These particles were then transferred to cryoSPARC for another round of ab initio reconstructions and 3D refinement.

### CryoDRGN classification

To identify conformational and compositional variability, volumes from the non-uniform refinement in cryoSPARC were further subjected to cryoDRGN^65,66^. First, the particles were binned from 352 pixels to 128 pixels (3.19 or 2.45 Å per pixel final pixel size) and used for training an 8-dimensional latent variable model with 3 hidden layers and 128 nodes in the encoder and decoder networks. The latent space was visualized with the analyze function on epoch 50, and clusters were extracted using *k*-means analysis. The particles from the cluster that yielded the best 3D refinement were moved forward for one more round of training, with binning to 256 pixels (1.595 or 1.22 Å per pixel final pixel size) and training with 3 hidden layers and 1,024 nodes in the networks. CryoDRGN landscape analysis was performed on the latent space, and the structures representing the center of each cluster were manually inspected. Finally, good particles selected in cryoDRGN were extracted and transferred to cryoSPARC for another round of ab initio reconstructions and 3D refinement.

### Model building and refinement

Local refinement, CTF, and de-focus optimizations were used to improve the overall resolution of each map. The individual domains (AAA, AAA2, AAA3, AAA4, linker, and different stalk helices) from yeast dynein models PDB: 7MGM and 5VH9, were docked, and rigid body fit into the different cryo-EM maps using UCSF Chimera X to build uniform models^67^. Subsequent rigid body and refinement was carried out using Phenix real space refine^68^. For all models the discrepancies between the model and map were fixed manually in COOT and then refined using a combination of Phenix real space refine and Rosetta Relax (v.13)^69,70^. For the 0.5 min dataset collected in the absence of Lis1, one model was built for the highest resolution density map (S1), given that the additional straight and intermediate state maps were derived from particles in S1 and were at a much lower resolution.

### ATPase assays

ATPase assays were performed using EnzChek phosphate kit (Thermo Fisher), as previously described^60^. The proteins for the final reaction mixtures contained 20 nM dynein monomer or 20 nM dynein–GST dimer and Lis1 at 0, 50, 100, 150, 200 or 250 nM, 2 mM Mg-ATP, 200 mM MESG (2-amino-6-mercapto-7methyl purine ribose), 1 U ml^–1^ purine nucleoside phosphorylate, and assay buffer (30 mM HEPES (pH 7.4), 50 mM potassium acetate, 2 mM magnesium acetate, 1 mM EGTA, 1 mM DTT). The final reaction signal was read at 360 nm every 10 s for 10 min on a Biotek Citation 5 plate reader.

### Molecular dynamics simulations

Four simulation systems were built based on the highest-resolution cryo-EM models obtained at 30-min time point (bent linker with 1× Lis1 bound (B7), intermediate linker with 2× Lis1 bound (I7), intermediate linker with 1×is1 bound (I6), and intermediate linker with no Lis1 bound (I4)). The linker region for each model was extended to start at residue 1354. For the I6 and I7 intermediate linkers, the linker position was determined from maps obtained from cryoDRGN heterogeneity analysis (Fig. 5c and Extended Data Fig. 2c). Each model was solvated in a water box containing 150 mM NaCl. All the MD simulations were performed using the GPU-accelerated version of Amber18 with the ff14SB force field^71,72^. Energy minimization was conducted with the protein atoms’ positions constrained by harmonic potentials with a spring constant of 10 kcal per mol Å^2^. With the same positional constraints, a 40-ns equilibration simulation was performed at 300 K, gradually reducing the spring constant from 10 to 0.01 kcal per mol Å^2^. Langevin dynamics with a friction coefficient of 1 ps^−1^ was applied to maintain a constant temperature. Particle Mesh Ewald was used for full-system periodic electrostatics, and a 9-Å cutoff was applied to Lennard–Jones interactions^73^. Bonds involving hydrogen atoms were constrained using the SHAKE algorithm^74^. Next, Gaussian Accelerated Molecular Dynamics (GaMD) was employed to enhance the sampling of protein conformational dynamics^75^. For each system, 20 independent runs were launched, each comprising a 40-ns conventional MD stage, a 20-ns GaMD equilibration stage, and a 100-ns GaMD production stage. The conventional MD stage was used to gather statistics for calculating initial GaMD acceleration parameters. During the GaMD stages, both total potential energy boost and dihedral energy boost were applied to the system, each with an upper limit of 4 kcal mol^–1^ for the s.d. (for accurate reweighting). For each system, the accumulated GaMD trajectories analyzed totaled 2 μs. The probability density distributions shown in Fig. 6 were obtained using the reweighting approach^76^.

### Statistical analysis

Data were analyzed with Microsoft Excel, and statistical tests were conducted using GraphPad Prism 9. The exact *n* values, evaluations of statistical significance, *P* values, and specific statistical analyses are described in the corresponding figures and figure legends.

### Reporting summary

Further information on research design is available in the Nature Portfolio Reporting Summary linked to this article.

## Online content

Any methods, additional references, Nature Portfolio reporting summaries, source data, extended data, supplementary information, acknowledgements, peer review information; details of author contributions and competing interests; and statements of data and code availability are available at 10.1038/s41594-025-01558-w.

## Supplementary information

Reporting Summary

Supplementary Video 1 The video shows a morph between different cryo-EM densities identified in the dynein and Lis1 dataset incubated for 0.5 mins with ATP.

## Source data

Source Data Fig. 1 Source data for Fig. 1.

Source Data Fig. 4 Source data for Fig. 4.

Source Data Fig. 5 Statistical source data for Fig. 5h,i.

Source Data Fig. 6 Source data for Fig. 6.

Source Data Extended Data Fig. 5 Source data for Extended Data Fig. 5.

## Figures and Tables

**Extended Data Table 1:**
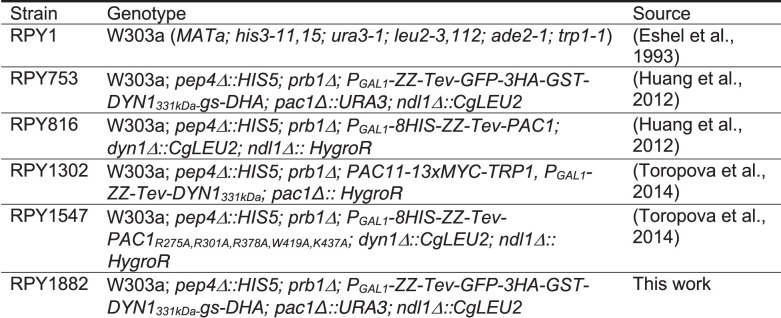
*S. cerevisiae* strains used in this study

## Data Availability

Cryo-EM maps and atomic coordinates have been deposited in the Electron Microscopy Data Bank under accession codes: S1, EMD-46919; S2, EMD-46897; S3, EMD-46938; S4, EMD-46953; I1, EMD-47019; I2, EMD-46958; I3, EMD-46941; I4, EMD-46954; I5, EMD-46974; I6, EMD-46972; I7, EMD-46975; B1, EMD-46962; B2, EMD-46940; B3,EMD-46942; B4, EMD-46935; B5, EMD-46959; B6, EMD-47033; B7, EMD-47026; and B8, EMD-47032. They have also been deposited in the Protein Data Bank under accession codes: S1, 9DIU; S2, 9DI3; S3, 9DJU; S4, 9DKD; I1, 9DMW; I2, 9DKH; I3, 9DJZ; I4, 9DKE; I5, 9DLD; I6, 9DKX; I7, 9DLE; B1, 9DKM; B2, 9DJY; B3, 9DK0; B4, 9DJ7; B5, 9DKJ; B6, 9DNB; B7, 9DN5; and B8, 9DN7. Unprocessed micrographs are deposited to EMPIAR under the following accession numbers: EMPIAR-12618, EMPIAR-12640, EMPIAR-12638, and EMPIAR-12584. Data and materials can be obtained from the corresponding authors upon request. Source data are provided with this paper.
