# Supplementary material for: Multiple steps of dynein activation by Lis1 visualized by cryo-EM
Source: Nat Struct Mol Biol. 2025 May 23;32(8):1434–44. doi: 10.1038/s41594-025-01558-w (PMC12350149; doi:10.1038/s41594-025-01558-w)
Supplement: Supplementary file 1 — Reporting Summary [file 41594_2025_1558_MOESM1_ESM.pdf]

nature portfolio

Corresponding author(s): Andres E. Leschziner and Agnieszka A. Kendrick

Last updated by author(s): 02/06/2025

Reporting Summary

Nature Portfolio wishes to improve the reproducibility of the work that we publish. This form provides structure for consistency and transparency in reporting. For further information on Nature Portfolio policies, see our Editorial Policies and the Editorial Policy Checklist.

Statistics

For all statistical analyses, confirm that the following items are present in the figure legend, table legend, main text, or Methods section.

n/a	Confirmed	
☐	☒	The exact sample size (*n*) for each experimental group/condition, given as a discrete number and unit of measurement
☐	☒	A statement on whether measurements were taken from distinct samples or whether the same sample was measured repeatedly
☐	☒	The statistical test(s) used AND whether they are one- or two-sided<br>*Only common tests should be described solely by name; describe more complex techniques in the Methods section.*
☒	☐	A description of all covariates tested
☐	☒	A description of any assumptions or corrections, such as tests of normality and adjustment for multiple comparisons
☐	☒	A full description of the statistical parameters including central tendency (e.g. means) or other basic estimates (e.g. regression coefficient) AND variation (e.g. standard deviation) or associated estimates of uncertainty (e.g. confidence intervals)
☐	☒	For null hypothesis testing, the test statistic (e.g. *F*, *t*, *r*) with confidence intervals, effect sizes, degrees of freedom and *P* value noted<br>*Give P values as exact values whenever suitable.*
☒	☐	For Bayesian analysis, information on the choice of priors and Markov chain Monte Carlo settings
☒	☐	For hierarchical and complex designs, identification of the appropriate level for tests and full reporting of outcomes
☒	☐	Estimates of effect sizes (e.g. Cohen's *d*, Pearson's *r*), indicating how they were calculated

Our web collection on statistics for biologists contains articles on many of the points above.

Software and code

Policy information about availability of computer code

Data collection	Cryo-EM data was collected on a Talos Arctica and Titan Krios (FEI), where automated collection was executed by Leginon or EPU, respectively. The ATPases assays were read on a Biotek Citation 5 plate reader.
Data analysis	We used Excel (Microsoft) and Prism v9 (Graphpad) for data analysis and plotting. Structure images and movie were generated with UCSF Chimera X1.7 and 1.8. Structural data were processed using UCSF MotionCor2, Relion 3.0, TOPAZ, cryoSPARC, COOT6, CTFFIND4, and Rosetta Relax (v.13). Illustrations were made in Illustrator CC (Adobe).

For manuscripts utilizing custom algorithms or software that are central to the research but not yet described in published literature, software must be made available to editors and reviewers. We strongly encourage code deposition in a community repository (e.g. GitHub). See the Nature Portfolio guidelines for submitting code & software for further information.

Data

Policy information about availability of data

All manuscripts must include a data availability statement. This statement should provide the following information, where applicable:

- Accession codes, unique identifiers, or web links for publicly available datasets
- A description of any restrictions on data availability
- For clinical datasets or third party data, please ensure that the statement adheres to our policy

Cryo-EM maps and atomic coordinates have been deposited in the Electron Microscopy Data Bank under accession codes: S1 – EMD-46919, S2 – EMD-46897, S3 –

EMD-46938, S4 – EMD-46953, I1 – EMD-47019, I2 – EMD-46958, I3 – EMD-46941, I4 – EMD-46954, I5 – EMD-46974, I6 – EMD-46972, I7 – EMD-46975, B1 – EMD-46935, B2 – EMD-46940, B3 – EMD-46942, B4 – EMD-46919, B5 – EMD-46959, B6 – EMD-47033, B7 – EMD-47026, B8 – EMD-47032 and in the Protein Data Bank under accession codes: S1 – 9DIU, S2 – 9DI3, S3 – 9DJU, S4 – 9DKD, I1 – 9DMW, I2 – 9DKH, I3 – 9DJZ, I4 – 9DKE, I5 – 9DLD, I6 – 9DKX, I7 – 9DLE, B1 – 9DKM, B2 – 9DJY, B3 – 9DK0, B4 – 9DJ7, B5 – 9DKJ, B6 – 9DNB, B7 – 9DN5, B8 – 9DN7. Unprocessed micrographs are deposited to EMPIAR under the following accession numbers: EMPIAR-12618, EMPIAR-12640, EMPIAR-12638 and EMPIAR-12584. Source data are available with the manuscript online. Data and materials can be obtained from the corresponding authors upon request.

Research involving human participants, their data, or biological material

Policy information about studies with human participants or human data. See also policy information about sex, gender (identity/presentation), and sexual orientation and race, ethnicity and racism.

Reporting on sex and gender	n/a
Reporting on race, ethnicity, or other socially relevant groupings	n/a
Population characteristics	n/a
Recruitment	n/a
Ethics oversight	n/a

Note that full information on the approval of the study protocol must also be provided in the manuscript.

Field-specific reporting

Please select the one below that is the best fit for your research. If you are not sure, read the appropriate sections before making your selection.

☒ Life sciences ☐ Behavioural & social sciences ☐ Ecological, evolutionary & environmental sciences

For a reference copy of the document with all sections, see nature.com/documents/nr-reporting-summary-flat.pdf

Life sciences study design

All studies must disclose on these points even when the disclosure is negative.

Sample size	No statistical tests were performed to determine sample size. Sample sizes were chosen so as to provide confidence and statistical power to our experimental findings. Each experiment was repeated at least three independent times. All data points collected befitting the criteria outlined in our materials and methods were included in our analysis.
Data exclusions	Data was not excluded from analysis.
Replication	Experiments were repeated three times in over two years and with different protein preparations. All replicates produced consistent results.
Randomization	Randomization is not relevant for this study because mutants were created and tested, rather than having experimental conditions assigned to different samples.
Blinding	Data was not blinded. This was because we thought that blinding was not necessary to avoid bias, because results have been reproduced independently by two different investigators, using different protein preparations, over a period of two years, and yielding consistent results.

Reporting for specific materials, systems and methods

We require information from authors about some types of materials, experimental systems and methods used in many studies. Here, indicate whether each material, system or method listed is relevant to your study. If you are not sure if a list item applies to your research, read the appropriate section before selecting a response.

Materials & experimental systems

n/a	Involved in the study
☒	☐ Antibodies
☒	☐ Eukaryotic cell lines
☒	☐ Palaeontology and archaeology
☒	☐ Animals and other organisms
☒	☐ Clinical data
☒	☐ Dual use research of concern
☒	☐ Plants

Methods

n/a	Involved in the study
☒	☐ ChIP-seq
☒	☐ Flow cytometry
☒	☐ MRI-based neuroimaging

Plants

Seed stocks: n/a

Novel plant genotypes: n/a

Authentication: n/a
